# Notes and Notices

**Published:** 1897-04

**Authors:** 


### NOTES AND NOTICES.

THE Southwestern Homeopathic Medical College, of Louisville, Kentucky, held its annual commencement on Tuesday afternoon, April 6th. The editor of THE HOMEOPATHIC PHYSICIAN desires to make this acknowledgment of the politeness of an invitation to the ceremonies.

ANTI-TOXINE KILLED HIM.—St. Louis, Mo., April 8th.—James M. Williamson, fifteen years old, died at the Christian Orphans' Home under circumstances most remarkable from a medical viewpoint. He was given an immunizing injection of anti-toxine for the purpose of preventing diphtheria. Forty minutes later the boy was dead. The news struck like a thunderbolt in the College of Physicians and Surgeons. An autopsy was ordered, in which half a dozen learned medicos took part.

After it was over they held a long consultation, and came out with the verdict, "Death from heart failure."—*Evening Bulletin*, Philada., April 8th, 1897.

HAS A MOON ON HIS NECK.—The queerest birthmark which the authorities tell anything about is on the back of Joseph Rotherman's neck. Joseph is a resident of Connellsville, Pa., and his odd mark is a moon. When the moon is new the mark is hardly noticeable, but by the end of the first quarter it is an angry-looking crescent, daily increasing in size. When the moon is at the full Rotherman's birthmark has also attained its maximum size. It decreases as the moon wanes, and by the time of old or new moon is again scarcely noticeable.—*St. Louis Republic.*

DOCTOR'S WIFE—"She will make a charming little widow. Why should you be discouraged? I assure you that he is lost—my husband is treating him."—*La Caricature.*

"WHAT you need is a warm climate, Mr. Grumpey," said the doctor in his most persuasive tone.

"I guess you'll get me there all right enough," was the ungracious response.—*Detroit Free Press.*

Dr. Arthur G. Allan has removed his office to 67 West Forty-ninth Street, where he may be consulted between 3 and 5 P. M. daily except Sundays. Dr. Allan may also be seen at his residence at the Hotel Imperial, Broadway and Thirty-second Street, before 10 A. M. Telephone No. 1247—Thirty-eighth Street.

Meeting of American Medical Publishers' Association.—The Fourth Annual Meeting of the American Medical Publishers' Association will be held in Philadelphia, on Monday, May 31st, 1897 (the day preceding the meeting of the American Medical Association). Editors and publishers, as well as every one interested in Medical Journalism, cordially invited to attend and participate in the deliberations. Several very excellent papers are already assured, but more are desired. In order to secure a place on the programme, contributors should send titles of their papers at once to the Secretary, Charles Wood Fassett, St. Joseph, Mo.

A Residence near the Seashore Advisable for Patients with Nervous Troubles.—It is a well-known fact that a residence in a low altitude near the seashore is beneficial and often curative with nervous diseases and certain forms of mental trouble. Dr. Given's Sanitarium, Stamford Hall for Nervous and Mental Diseases, at Stamford, Conn., affords home comforts and the special treatment each particular patient requires.

Hahnemann Medical College, Philadelphia.—The annual reunion and banquet of the Alumni Association of the Hahnemann Medical College, Philadelphia, will be held on Wednesday, May 12th, 1897. The business meeting will convene at 4.30 P. M. in Alumni Hall, Hahnemann Medical College, Broad Street above Race, Philadelphia, and the banquet will be held at 9.45 P. M. at the "Walton," southeast corner Broad and Locust Streets. The Trustees and Faculty of the College extend a cordial invitation to all the members of the Alumni and their friends to attend the Forty-eighth Annual Commencement, to be held on the same evening, at 8 o'clock, at the Academy of Music, southwest corner Broad and Locust Streets, Philadelphia. Banquet cards can be secured by notifying the Secretary. Requests received after Tuesday, May 11th, 1897, cannot be considered. W. W. Van Baun, M. D., Secretary, 1402 Spruce Street, Philadelphia.

The Hahnemann Medical Association of Iowa will hold its annual session at Council Bluffs, Iowa, May 12th, 13th, and 14th, 1897. An interesting programme is being prepared. Dr. T. L. Hazard, of Iowa City, Iowa, is the Secretary.

The Homeopathic Medical Society of Tennessee will meet at Nashville, May 19th and 20th, 1897. Arrangements are being made for a very successful meeting. It is hoped every member will be present, and that all the fraternity in the State who have not joined will do so then. The expenses are nominal. The benefits to be obtained are great, both individually and collectively, as Homeopathy has much to gain by concerted action. The sections with topics to be discussed will be published later. It is the wish of the Society, as expressed at its last meeting, that every paper under each bureau discuss the same topic in its different phases. It is hoped every physician will arrange his affairs so as to be present the entire time and make the sessions mutually profitable and pleasant. For particulars address Dr. Chas. G. Wilson, 203 Main Street, Clarksvile, Tenn.

The College of Physicians and Surgeons, of Chicago, has recently changed its name to The Medical School of the University of Illinois. Dr. Wm. Allen Pusey, 103 State Street, Chicago, is Secretary of the Faculty.

Dr. Catharine V. C. Scott, of San Francisco, California, has removed her office and residence from 727 Geary Street to 14 Grant Avenue, City of Paris Building, San Francisco. Her hours are 11 A. M. to 3 P. M., and 7 to 7.30 P. M., Sundays excepted. She makes a specialty of the diseases of women.

The Society of Homeopathicians will hold its next meeting at "The New Mathewson," Narragansett Pier, Rhode Island, June 22d, 23d, 24th, and 25th, 1897. For particulars address Dr. S. A. Kimball, Secretary, 124 Commonwealth Avenue, Boston, Mass.

Orificial Surgery and Gynecology.—The first *Eastern* annual course of instruction in Orificial Surgery will be delivered at the Muncie Seaside Sanatorium, Brooklyn, New York, commencing July 12th, and continuing one week, the sessions being four hours daily. This course will be conducted by Prof. E. H. Pratt, A. M., M. D., LL. D., assisted by E. Z. Cole, M. D., Professor of Surgery in Baltimore Medical College, and Drs. E. H. and L. H. Muncie, of Muncie Sanatorium, of Brooklyn. For terms apply to Drs. E. H. and L. H. Muncie, Muncie Sanatorium, Macon Street, cor. Marcy Avenue, Brooklyn, New York.

The Missouri State Medical Association will hold its fortieth annual meeting at the Century Theatre, St. Louis, Mo., May 18th, 19th, and 20th, 1897.

Denver Homeopathic Medical College and Hospital held its third annual commencement Friday evening, April 2d, 1897, at 7.30 o'clock, in Trinity Church, Denver. The Editor desires to acknowledge the courtesy of an invitation.

### LOCAL TREATMENT OF CHRONIC GASTRIC CATARRH—A CLINICAL LECTURE.

By J. M. G. Carter, M. D., Sc. D., Ph. D., Professor of Clinical and Preventive Medicine in the College of Physicians and Surgeons, Chicago, Fellow of the American Academy of Medicine, etc.

(Reprint from the *American Therapist*, January, 1897.)

Local treatment may be applied in any stage of chronic gastric catarrh; but it must be varied somewhat in the different stages. The grade of inflammation, its character and persistence, likewise may require some modification of the treatment.

*First Stage.*—During the incipiency of chronic gastritis, local treatment is not so essential except in bacterial cases, but is beneficial. It serves to modify the congestion when that is increased, and often allays dyspeptic symptoms even when they are more marked than usual. The use of warm water (105 degrees) with bicarbonate of sodium (three per cent.) for washing out the stomach is frequently very valuable to remove the tenacious mucus usually adhering to the gastric mucous membrane, in this condition, and interfering with the proper mixing of peptic fluid with the food. The patient may drink a glassful of the solution before meals or it may be introduced into the stomach through the tube. If the tube is used, the stomach should be filled before allowing any reflow. The cold douche with water at 80 degrees to 60 degrees is sometimes more grateful and helpful than the hot douche (110 degrees to 125 degrees). A continuous effect may be secured by using a double tube and permitting the inflow and outflow to progress simultaneously; but care should be taken to keep the stomach distended sufficiently to have the solution come in contact with the entire gastric surface. The soda solution dissolves the mucus and the stream washes it away. Weak soap-suds may be used with the tube for the same purpose. More satisfactory in many instances is the use of a solution of Hydrozone. A glassful (eight fluid ounces) of a two or three per cent. solution may be given half an hour before meals. If used as a douche with the tube a five per cent. solution is not too strong, and two quarts the minimum amount. These douchings may be given one to six or seven times a week, according to the requirements of the case, and are frequently all the treatment this stage of chronic gastritis demands, except what changes are necessary in the diet.

*Second Stage.*—The inflammatory process is fully developed in the second stage, and while there may be weeks or months when there is little if any suffering the treatment should be persistent. The cleansing of the gastric mucous membrane must be systematic and thorough. This is best accomplished with a solution of green soap or a five or eight per cent. solution of Hydrozone, introduced with the double tube. After first filling the stomach, inflowing and outflowing streams ought to remain about equal or the outflow may exceed the inflow, the distention of the stomach may be maintained by retarding the reflow when necessary. This process can be beneficially accomplished by driving the solution into the stomach under increased air pressure; but when the proper apparatus for this method is not at hand the siphoning method with the single tube does very well. For home treatment or when the tube cannot for any reason be used, a solution may be made for drinking. For this purpose a two or three per cent. solution of Hydrozone is prepared. The patient may take a glassful (8 oz.) half an hour before meal time. He should lie down at once, remain five minutes on the back, then turn on the right side, where he must remain during the remainder of the half-hour. While the patient is on the back the solution comes in contact with every portion of the gastric mucous membrane and turning to the right side facilitates the emptying of the stomach. By this process the offending mucus is dissolved and carried away and the organ is put into a proper condition to digest food. The use of Hydrozone has the additional advantage of checking the growth of the bacteria, and probably exhibits greater antiseptic properties than any other agent that can be used in the stomach with the same degree of safety. In obstinate cases this cleansing ought to precede every meal.

After the stomach is cleansed it should be treated with soothing, stimulating, and healing applications. There are many preparations which can be used, some of the best of which are glycerole of bismuth and eucalyptol, the essential oils and Glycozone. Boric acid in two or three per cent. solution as a wash with the tube is sometimes very valuable. The other agents mentioned may be used with a nebulizer, by means of which a vapor impregnated with the medicines can be passed into the stomach through a tube, the double tube being preferable. If it is not convenient to use a nebulizing apparatus, the glycerole mentioned, and especially Glycozone, may be administered by the mouth. In many cases, in fact, the latter mode of administering these agents is more desirable. These remedies encourage healing and materially enhance the patient's prospects of recovery. This is especially true in bacterial cases. When Hydrozone has been given before meals as already suggested for cleansing purposes, Glycozone may be adminstered in teaspoonful doses after meals with very satisfactory results. This line of treatment is frequently so successful that cases are temporarily relieved and possibly often a cure effected, particularly if the general treatment has been judiciously carried out.

If, for any reason, Glycozone cannot be employed the essential oils may be used. The oils of anise, peppermint, cubebs, and tar may be combined and used with a nebulizer as previously suggested. Although benefit may be derived from the administration of this combination, I prefer the Glycozone treatment. The use of hot water, 120 degrees or more, and the employment of cold water, 80 degrees to 40 degrees (F.) may give very happy results in certain severe cases.

*Third Stage.*—The condition referred to here is one of atrophy. The functions of absorption and motion may be fairly well performed. The chief difficulty then is with the digestion of proteids. The local treatment has two objects, mainly, although a third is sometimes in mind. The first object is the removal of *debris* and foreign material. The second is the cleansing of the mucous membrane and the destruction of micro-organisms and their removal in order that the intestines may not receive bacterial products from the stomach. The third object sometimes kept in view in the local treatment by douching is a degree of stimulation of the functions of motion and absorption and the tonic effect to the gastric walls which follows those washings. The first object is accomplished by the use of sterilized water or a three per cent. solution of sodium bicarbonate. Either tube may be used. The second object is effected by douching the walls with a green soap solution or a solution of Hydrozone. The latter agent in five per cent. solution as directed above gives very pleasing results. The third object may be secured by using hot or cold water for the douche.

100 State Street, Chicago.

